# Antifungal Activity of Natural Thymol: Advances on Molecular Mechanisms and Therapeutic Potential

**DOI:** 10.3390/biom16010149

**Published:** 2026-01-14

**Authors:** Chun Chen, Lu Liu, Shusheng Tang, Daowen Li, Chongshan Dai

**Affiliations:** 1 Technology Innovation Center for Food Safety Surveillance and Detection (Hainan), Sanya Institute of China Agricultural University, Sanya 572025, China; 2 State Key Laboratory of Veterinary Public Health and Safety, College of Veterinary Medicine, China Agricultural University, Beijing 100193, China; 3 Tianjin Key Laboratory of Agricultural Animal Breeding and Healthy Husbandry, College of Animal Science and Veterinary Medicine, Tianjin Agricultural University, Tianjin 300384, China

**Keywords:** thymol, antifungal activity, molecular mechanisms, safety, clinical applications

## Abstract

Currently, the increased incidence of invasive fungal infections globally is posing a significant challenge to public health. Due to drug resistance issues, the clinical efficacy of existing antifungal drugs is seriously insufficient, while new drug development progresses slowly. Consequently, there is an urgent need to discover and develop novel antifungal therapeutics. Natural products have the characteristics of wide sources and few adverse reactions and are one of the sources for developing antifungal drugs. Numerous studies have shown that many compounds isolated from plants and traditional Chinese medicine have antifungal activity and diverse antifungal mechanisms. Thymol, a monoterpene phenol compound from thyme (*Lamiaceae*), has multiple biological functions such as antibacterial, antioxidant, and anti-inflammatory. Recent research has found that thymol has strong antifungal activity, and its molecular mechanisms involve cell membrane rupture, interference with cell wall synthesis, disruption of mitochondrial function and energy metabolism, inhibition of biofilm, inhibition of virulence factor expression, inhibition of key enzymes, and induction of cell apoptosis. This review aimed to summarize the antifungal activity of thymol and the underlying molecular mechanisms, safety, and potential clinical applications. Emerging technologies in thymol delivery systems and future research directions are also discussed. The comprehensive analysis aims to provide a detailed understanding of fungal infections and the role of thymol in antifungal treatment, offering insights for further research and clinical practice.

## 1. Introduction

Fungal diseases are posing a significant threat to human and animal health [1,2,3]. In humans, invasive fungal infections have become increasingly common, especially in immunocompromised patients [4]. For instance, invasive pulmonary aspergillosis (IPA) is a severe manifestation of pulmonary aspergillosis. Among all clinical manifestations of pulmonary aspergillosis, IPA is the most acute presentation, caused by *Aspergillus* hyphae invading the pulmonary tissue [5]. In a study conducted at a Mexican hospital during the COVID-19 pre-vaccination era, the prevalence of possible COVID-19-associated pulmonary aspergillosis (CAPA) was 4.13% among 2080 individuals with severe COVID-19. All CAPA cases had a positive respiratory culture for *Aspergillus* species, with *Aspergillus fumigatus* being the most frequent isolate (64%) [6]. In organ transplant recipients, cryptococcosis is the third most commonly occurring invasive fungal disease [7]. Although kidney transplant recipients are at a relatively lower risk compared to other solid-organ transplant recipients, the mortality rates with cryptococcosis range from 10– to 25% and can be as high as 50% in those with central nervous system involvement [8]. In Japan, a nationwide observational study from 2015 to 2021 on disseminated cryptococcosis found that the age-adjusted incidence rate was higher in men, and the prevalence did not show a significant increase over the study period, with lower incidence observed in Northern Japan [9]. In animals, fungi can cause cutaneous mycoses, histoplasmosis, and coccidioidomycosis [10]. They can also contaminate animal feed via the toxic secondary metabolites, such as aflatoxins, T-2 toxin, and zearalenone, which can lead to mycotoxicosis [11,12,13]. Unfortunately, some fungal pathogens, such as *Candida*, *Cryptococcus*, and *Aspergillus*, exhibited potential resistance, resulting in a decreasing number of clinically available drugs [14,15,16]. Therefore, there is an urgent need to develop new antifungal drugs.

**Figure 1:**
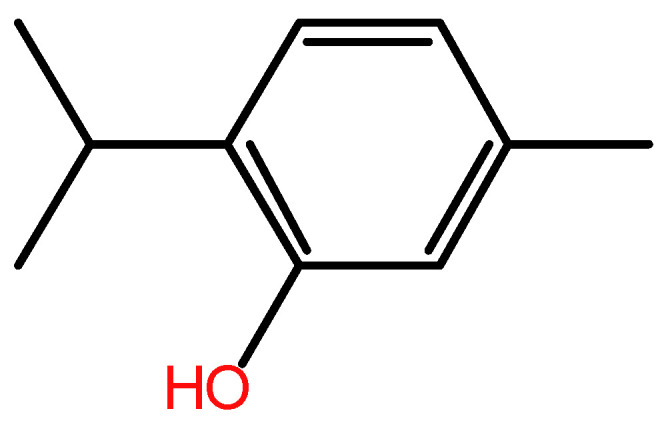
The chemical structure of thymol.

In recent decades, natural products have proven to be a diverse source of bioactive compounds with potential antifungal properties [17,18,19]. Multiple studies have shown that some natural products exhibited strong antifungal activity or reversed the resistance of some fungi to drugs, demonstrating potential clinical application value [20,21,22]. Thymol (the structure is shown in Figure 1) is a naturally occurring phenolic compound and can be found in multiple essential oils extracted from many plants, including oregano, thyme, sweet basil, black cumin, elsholtzia, and savory [23], which are widely distributed throughout China, Korea, and Europe [16]. Thymol has been reported to exhibit a broad spectrum of antifungal activities [24,25,26,27]. Importantly, thymol and its isomer carvacrol are the main active ingredients of these plant essential oils, and their compound proportion is high and accounts for up to about 20–60% [28,29]. In addition, some compounds, such as estragole, 1,8-cineole, terpineol-4, and γ-terpinene, found in these essential oils, also exhibited potent antifungal activity [30,31,32,33,34]. Previous studies have demonstrated that thymol has potent potential to inhibit biofilm formation of various fungal pathogens, such as *Fusarium* species and *Candida* species [35,36,37]. Gao et al. showed that the average median effective concentration (EC_50_) value of thymol for 59 *Fusarium graminearum* isolates was about 26.3 μg/mL, and its antifungicidal activity is mainly attributed to the induction of lipid peroxidation and the disruption of ergosterol biosynthesis [38]. Thymol, in combination with other active ingredients in these essential oils at a special proportion, exhibited potent additive or synergistic effects. For example, Radocchia et al. reported that the combination of thymol and carvacrol exhibited potent synergistic effect against *C. albicans* (FICI is 0.24) and *C. tropicalis* (FICI is 0.37) [39]. In addition, it also has strong biological activities such as anti-inflammatory, antioxidant, and immune regulation and has a certain tissue repair effect [27]. Wang et al. found that thymol supplementation can markedly improve the inflammatory response caused by *Aspergillus fumigatus* infection by inhibiting lectin-type oxidized LDL receptor 1 (LOX-1)/interleukin-1β (IL-1β) signaling pathway-mediated inflammation activation and reducing the recruitment of neutrophils and macrophages in a mouse model [40]. It was also reported that thymol, in combination with other antifungal agents, such as posaconazole, fluconazole, and nystatin, can effectively reduce biofilm formation and disrupt mature biofilms, thereby enhancing the susceptibility of *Candida* species [41,42,43,44]. Collectively, these studies underscore the potential of thymol as a versatile antifungal agent with applications in agriculture and medicine.

In the present study, we utilized keywords such as “thymol” or “thyme essential oil”, and “antifungal activity” to gather research on the antifungal activity of thymol and the underlying molecular mechanisms from databases such as Web of Science and PubMed, up to November 2025. The collected information was then summarized and discussed. Then, we summarized the antifungal activity of thymol and the underlying molecular mechanisms. Additionally, the safety, potential clinical applications, emerging technologies in thymol delivery systems, and future research directions were also discussed. We hope this review paper can provide a detailed understanding of fungal infections and the role of thymol in antifungal treatment, offering insights for further research and clinical practice.

## 2. Epidemiology of Fungal Infections and Thymol’s Role

### 2.1. Prevalence and Impact of Fungal Infections

Fungal infections have a significant global impact on public health, affecting various populations and causing a wide range of diseases [45]. In Greece, an estimate of the burden of serious fungal diseases showed that annually, out of a population of 10.8 million, about 1.79 cases per 100,000 individuals suffer from serious fungal diseases [46]. Approximately 1.93% of the population in Thailand (an estimated 1,254,562 people) is affected by serious fungal infections, including invasive aspergillosis and candidiasis [47]. In the Czech Republic, around 176,000 individuals (1.67% of the population) suffer from severe fungal infections each year, primarily manifesting as recurrent vaginitis and allergic respiratory diseases [48]. Additionally, nearly one million people in Ukraine are affected by fungal diseases annually [49]. Fungal infections range from mild superficial conditions (like athlete’s foot) to severe systemic diseases (such as invasive aspergillosis or cryptococcosis). Among these, the following five globally prevalent fungal diseases cause significant morbidity in both humans and animals, i.e., candidiasis, aspergillosis, cryptococcosis, dermatophytosis, and Pneumocystis pneumonia (Table 1). Candidiasis is caused by fungi of the *Candida genus*, most commonly *Candida albicans*, though other clinically relevant species such as *Candida glabrata*, *Candida parapsilosis*, *Candida tropicalis*, and *Candida krusei* are increasingly significant in clinical settings [50,51]. Both humans and various animal species are susceptible, particularly immunocompromised individuals. The disease manifests as superficial infections, including cutaneous candidiasis, oral thrush, and vulvovaginal candidiasis, or as invasive infections that may lead to candidemia and multiorgan dissemination [52]. Aspergillosis refers to infections or allergic conditions caused by fungi of the *Aspergillus genus*, most commonly *Aspergillus fumigatus* [53,54]. This pathogen is highly transmissible, primarily via airborne spores, and manifests in several clinical forms including invasive aspergillosis, chronic necrotizing pulmonary aspergillosis, allergic bronchopulmonary aspergillosis, aspergilloma (fungus ball), as well as localized infections such as keratitis and otitis externa [55]. Cryptococcosis is a fungal disease caused by species of the *Cryptococcus genus*, primarily *Cryptococcus neoformans* and *Cryptococcus gattii*, and infection typically occurs through inhalation of airborne cryptococcal spores in humans and animals [56,57]. Cryptococcal meningitis represents the most significant clinical manifestation, particularly in individuals with HIV infection [58]. Dermatophytosis refers to a group of superficial fungal infections affecting keratinized tissues (skin, hair, and nails), primarily caused by dermatophytes belonging to the genera *Trichophyton*, *Microsporum*, and *Epidermophyton* [59,60]. These fungi are widely prevalent across the globe and exhibit zoonotic potential, enabling transmission between humans and animals [61]. Infections may involve virtually any skin surface and hair [62,63]. Pneumocystis pneumonia (PCP) is a pulmonary infection caused by the fungus *Pneumocystis jirovecii* [64,65]. Transmitted via the respiratory route, the pathogen exhibits low-level circulation in human populations and primarily colonizes the human alveoli [65]. In developing countries, PCP remains a leading cause of mortality among individuals with HIV/AIDS [66,67]. In recent years, the emergence of resistance in *Aspergillus* and *Candida* spp. to azole drugs has been a growing issue [68]. This underscores the urgent need for novel antifungal agents.

Thymol has proven effective in diverse fungal infection scenarios, encompassing both in vitro and in vivo settings. Research highlights thymol’s robust antibiofilm capabilities against *Cryptococcus neoformans* and *Cryptococcus laurentii*, where it curtails growth, biofilm development, and established biofilms in these fungi [69]. In animal-based investigations, thymol also exhibits significant potential. For example, in studies on Nile tilapia, initial in vitro work demonstrated thymol’s antifungal properties, alongside other essential oils, targeting yeasts derived from infected fish. Subsequent in vivo experiments showed that incorporating thymol into diets markedly improved fish growth metrics, immune function, and survival rates (90%) when challenged with *Cryptococcus uniguttulatus* [70]. These outcomes indicate thymol’s utility as a potent agent for combating fungal infections in human and animal contexts, emphasizing its prospects for refinement as an antifungal therapy.

**Table 1:** Top 5 globally prevalent fungal diseases.

Disease	Principal Pathogen(s)	Host Range	Epidemiological Features	References
Candidiasis	*Candida albicans*, etc.	Humans and various animals	High risk in immunocompromised individuals	[50,51,52,71,72]
Aspergillosis	*Aspergillus fumigatus*	Humans, birds	Airborne transmission	[53,54,73]
Cryptococcosis	*Cryptococcus neoformans*/*C. gattii*	Humans, cats, dogs	Associated with environmental exposure; high risk in patients with HIV	[55,56,57,58,74,75]
Dermatophytosis	*Trichophyton*, *Microsporum*, etc.	Humans, pets, livestock	More prevalent in moist, crowded environments	[59,60,61,62,63]
Pneumocystis pneumonia	*Pneumocystis jirovecii*	Humans	High risk in patients with HIV/AIDS	[64,65,66]

### 2.2. An Overview of Thymol’s Antifungal Activity

Thymol exhibits broad-spectrum antifungal activity against a variety of fungi, with its effective concentration and mechanism of action varying by species. Against phytopathogenic fungi, thymol shows the highest activity against *Botrytis cinerea*, with a MIC as low as 12.30 µg/mL, primarily by disrupting cell membrane integrity [76]. The half-maximal effective concentrations EC_50_ against *Fusarium graminearum* and *Batrachochytrium salamandrivorans* are 26.3 µg/mL and 25 µg/mL [38,77], respectively, involving mechanisms such as the induction of lipid peroxidation and interference with ergosterol biosynthesis. For *Trichophyton rubrum*, the MIC of thymol is 32 µg/mL, while derivatives can significantly enhance the activity, reducing the MIC to 4 µg/mL [78]. It also demonstrates moderate to strong inhibitory effects against other common pathogens, such as *Alternaria alternata* (MIC = 250 µg/mL) [79] and *Rhizoctonia solani* (MIC = 250–500 µg/mL) [80], achieved by damaging the cell wall and cell membrane. Against human and animal pathogenic yeasts, thymol is effective against various *Candida species*. Notably, it exhibits activity against non-albicans *Candida species*, including *Candida glabrata*, *Candida krusei*, and *Candida tropicalis*, with MICs ranging from 32 to 128 µg/mL, and effectively disrupts biofilm formation [81]. Importantly, it retains activity against fluconazole-resistant strains of *Candida dubliniensis* (MIC = 64 µg/mL) [81]. For *Candida albicans*, the MIC range is broader (32–256 µg/mL) [81], with mechanisms including the induction of reactive oxygen species (ROS) burst, inhibition of efflux pumps [82], and suppression of ergosterol biosynthesis [81]. Against *Malassezia pachydermatis*, thymol shows relatively lower activity (MIC = 800 µg/mL), where its hydrophobicity facilitates disruption of ion gradients across the cell membrane [83].

The core antifungal mechanisms of thymol are highly focused on disrupting cell membrane integrity (e.g., pore formation, inhibition of ergosterol biosynthesis) and inducing oxidative stress (ROS accumulation). Additionally, it interferes with cell wall metabolism (e.g., inhibition of chitin synthesis [84], activation of cell wall-degrading enzymes [85]) and ultimately leads to cell death by disrupting ion homeostasis and energy metabolism [86]. Formulation improvements, such as nanoparticle encapsulation, can further enhance its targeting and antifungal efficacy [76], demonstrating its potential against antifungal drug-resistant strains. A systematic summary of thymol on antifungal activity and potential effects is presented in Table 2.

**Table 2:** Antifungal activity and mechanisms of action of thymol.

Fungal Species	MIC/EC_50_ (µg/mL)	Effects	References
*Alternaria alternata*	250	It damages the cell wall and cell membrane, leading to cytoplasmic disorganization and organelle destruction; inhibits spore germination, hyphal growth, and early infection structure formation.	[79]
*Aspergillus flavus*	80	It induces ROS burst and NO production; the combined action of ROS and NO leads to spore lysis and cell death.	[87]
*Batrachochytrium salamandrivorans*	25	It interferes with ergosterol biosynthesis, increases cell membrane permeability, and degrades cell function.	[77]
*Botrytis aclada*	30 (Fumigation)	It likely acts by inhibiting the synthesis of chitin or β-1,3-glucan (fungal cell wall components).	[84]
*Botrytis cinerea*	12.30 (Thymol) 9.90 (Chitosan-Thymol NPs)	It disrupts cell membrane integrity; nanoparticles of thymol are pH-responsive and enable intelligent release in the acidic environment created by the pathogen, then enhance targeted inhibition.	[76]
*Botrytis cinerea*	65	It disrupts cell membrane integrity, leading to leakage of cellular contents, decrease in extracellular pH, and increase in conductivity, finally causing hyphal deformation, shrinkage, and rupture.	[88]
*Candida albicans*	32–256	It disrupts cell membrane integrity, inhibits ergosterol synthesis, induces ROS generation, causing oxidative damage, and significantly damages mature biofilms. It also inhibits efflux pump activity and exhibits synergy with fluconazole.	[81,82]
*Candida dubliniensis*	64	It is effective against fluconazole-resistant strains via disrupting cell membrane structure.	[81]
*Candida glabrata* *Candida krusei* *Candida tropicalis*	32–128	It effectively disturbs the growth of both planktonic cells and biofilms.	[81]
*Colletotrichum chrysophillum* *Colletotrichum nymphaeae*	100	It disrupts cell membrane integrity based on the membrane-interfering properties of monoterpenes.	[89]
*Fusarium graminearum*	EC_50_ = 26.3	It can induce lipid peroxidation, disrupt ergosterol biosynthesis, cause cell membrane damage, and affect hyphal morphology and conidia production.	[38]
*Fusarium oxysporum f.* sp. *niveum*	EC_50_ = 80	It inhibits cell wall synthesis and activates cell wall-degrading enzymes. It can also disrupt membrane integrity, induce the production of ROS, and disturb TCA cycle and fatty acid metabolism.	[85]
*Fusarium tricinctum*	90	It destroys cell ultrastructure, disrupts plasma membrane integrity, transiently increases energy metabolism, and induces lipid peroxidation.	[36]
*Malassezia pachydermatis*	800	Hydrophobicity allows distribution into the cell membrane; free hydroxyl group disrupts ion gradients, leading to membrane dysfunction.	[83]
*Rhizoctonia solani*	250–500	It can cause severe hyphal malformation, shrinkage, inhibits cell wall-degrading enzyme activity, and disrupt cell membrane integrity.	[80]
*Trichophyton rubrum*	32 (Thymol) 4 (Derivative)	It disrupts the structure and function of the fungal cell membrane via inhibiting the ergosterol biosynthesis pathway.	[78]
*Zygosaccharomyces rouxii*	62.5	It disrupts cell membrane integrity, causing membrane perforation, membrane potential hyperpolarization, and the decrease in intracellular ATP and pH, followed by disrupting cell homeostasis and inducing cell death.	[86]

## 3. The Underlying Molecular Mechanisms

### 3.1. Disruption of the Fungal Cell Membrane and Mitochondrial Function

Thymol’s antifungal activity is partly attributed to its ability to disrupt the fungal cell membrane. Similarly to other membrane-active compounds, thymol can interact with the fungal cell membrane, leading to changes in its permeability and integrity [90]. Thymol primarily acts on the cell membrane surface. It can interact with the polar headgroups (especially the phosphate groups) of negatively charged phospholipids via hydrogen bonding or electrostatic interactions. This leads to monolayer expansion, reduced elasticity, and the induction of local aggregation. Concurrently, thymol mildly perturbs the packing of the alkyl chains, increasing their disorder and thereby enhancing membrane fluidity of fungal cells [91,92]. Collectively, these structural alterations compromise membrane stability and function, which may underlie its associated biological activities. For example, Kim et al. demonstrated that thymol induces the generation of ROS, which disrupts the cell membrane integrity of *Phytophthora quercina* and *Agrobacterium tumefaciens*, ultimately leading to cell death [93]. Treatment with the γ-cyclodextrin inclusion complex of thymol (γ-CDTL) for one hour resulted in disruption of cell membrane integrity in *Penicillium* spp. [94]. Fungal mitochondrial function can be disrupted by thymol, which may contribute to its antifungal activity. In many fungal pathogens, disruption of mitochondrial function can lead to cell death. Exposure of *Zygosaccharomyces rouxii* to subinhibitory concentrations of thymol induced intracellular accumulation of ROS, elevated Ca^2+^ levels, and decreased mitochondrial membrane potential (MMP), demonstrating its disruptive effect on the cell membrane [95]. Wang et al. revealed that thymol treatment induces increased membrane permeability in *Zygosaccharomyces rouxii*, leading to cytoplasmic leakage and mitochondrial membrane hyperpolarization, accompanied by the formation of pores in the cell membrane [86]. Treatment with thymol resulted in a significant increase in relative electrical conductivity and enhanced cell membrane permeability in *Fusarium graminearum*, indicating that thymol induces damage to the cell membrane [38]. In addition, some studies have shown that compounds affecting the ergosterol biosynthesis in the fungal cell membrane can have antifungal effects. Ergosterol is a key component of the fungal cell membrane, and disruption of its synthesis or interaction with it can lead to membrane dysfunction. Thymol interferes with the ergosterol biosynthesis or directly interacts with the membrane components, causing membrane disruption. This is supported by the fact that many natural products with antifungal activity target the fungal cell membrane, and thymol, being a natural monoterpene phenol, shares similar mechanisms of action [96]. Thymol inhibits ergosterol biosynthesis in *Fusarium graminearum* by downregulating the expression of genes FGSG_02771, FGSG_09031, and FGSG_11044 [38]. Thymol targets the mitochondria of fungal cells, inducing a reduction in MMP and an increase in ROS production. These effects lead to disruption of the cell membrane structure and inhibition of sterol lactone biosynthesis, thereby impairing cellular energy metabolism and ultimately resulting in cell death.

### 3.2. Inhibition of Fungal Cell Wall Activity

The fungal cell wall is a crucial target for antifungal agents [97], and thymol exerts its antifungal effects by interfering with cell wall synthesis or integrity. Thymol inhibits the growth of *Aspergillus niger* by increasing cell wall permeability [98]. Thymol may act in a similar way with poacic acid, a plant-derived antifungal agent, which can target β-1,3-glucan, a major component of the fungal cell wall [99]. It can also either directly interact with cell wall components or interfere with the enzymes involved in cell wall synthesis [94]. For example, it was reported that thymol can inhibit fungal growth in *Alternaria alternata* by simultaneously disrupting the cell wall and plasma membrane and damaging organelles within the cytoplasm [79]. Furthermore, Zhang et al. employed RNA-Seq analysis to investigate changes in gene expression in Fusarium species following thymol treatment. They observed significant downregulation in the majority of genes associated with glycosphingolipid biosynthesis and sphingolipid metabolism. In contrast, genes involved in chitin biosynthesis and cell wall remodeling were upregulated. These results demonstrate that thymol exerts its antifungal effects by suppressing genes critical to cell wall and membrane synthesis, inducing ROS accumulation, and ultimately disrupting cell wall integrity [85].

### 3.3. Thymol Induces Apoptosis in Fungal Cells

Apoptosis is a form of programmed cell death [100,101]. Previous studies have demonstrated that thymol can induce both intrinsic and extrinsic apoptotic pathways in fungi. Hu et al. demonstrated that exposure of *Aspergillus flavus* conidia to thymol (200 μg/mL) for 6 h induced chromatin condensation, DNA fragmentation, reduced MMP, and activation of caspase-9, indicating thymol-triggered caspase-dependent apoptosis. The antifungal effect was rescued by 4-aminopyridine, confirming that thymol targets potassium channel subunit beta (KCNAB) to induce K^+^ efflux, which subsequently promotes ROS accumulation and ultimately leads to apoptotic cell death in *Aspergillus* conidia. Treatment of *Zygosaccharomyces rouxii* with thymol resulted in increased cytochrome c release, caspase activation, and elevated DNA fragmentation. These effects were accompanied by upregulation of pro-apoptotic factors including yeast caspase-1 (YCA1), dynamin-1 (DNM1), nuclease-1 (NUC1), NADH dehydrogenase 1 subunit (NDI1), and microtubule and mitochondria interacting protein (MMI1), along with downregulation of anti-apoptotic regulators such as fission-1 (FIS1) and cell division control protein-48 (CDC48), collectively indicating thymol-induced apoptosis in *Zygosaccharomyces rouxii* [95]. Darvishi E et al. demonstrated that thymol, at a subinhibitory concentration of 50 μg/mL, accelerates telomere shortening by inhibiting expressed sequence tag-2 (EST2) transcription and thereby blocking telomerase activity, ultimately promoting fungal cellular senescence and apoptosis [102].

### 3.4. The Multifaceted Therapeutic Contributions of Thymol’s Antioxidant and Anti-Inflammatory Properties in Antifungal Applications

In addition to its antifungal properties, thymol’s antioxidant and anti-inflammatory activities significantly contribute to its therapeutic potential. The antioxidant activity of thymol is crucial in mitigating oxidative stress, a common pathway in fungal pathogenesis. Previous studies provide evidence of thymol’s ability to reduce ROS production and lipid peroxidation, thereby protecting cells from oxidative damage [103]. This antioxidant effect is complemented by thymol’s anti-inflammatory properties, which involve the modulation of key inflammatory pathways such as MAPK and NF-κB, leading to a reduction in pro-inflammatory cytokines [104,105]. These combined effects not only enhance thymol’s antifungal efficacy but also contribute to its role in reducing inflammation and oxidative stress associated with fungal infections. The integration of thymol’s antioxidant and anti-inflammatory activities into its antifungal therapy underscores the importance of a holistic approach to treating fungal infections. By targeting multiple pathways, thymol not only inhibits fungal growth but also alleviates the inflammatory and oxidative stress responses that often accompany such infections. This multifaceted approach is particularly relevant in the context of increasing antifungal resistance, as it offers a natural and potentially more effective alternative to synthetic antifungal agents [26].

**Figure 2:**
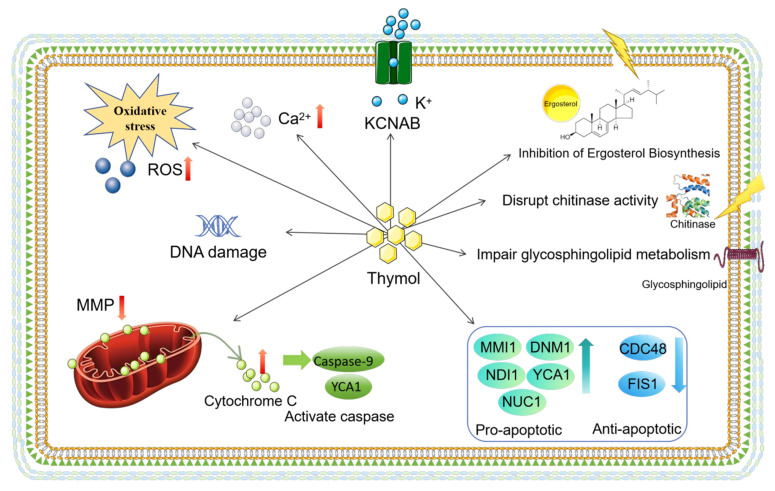
Thymol exerts its antifungal effect through multi-target synergistic actions. Firstly, it disrupts the peripheral cellular structures by increasing cell wall permeability, inhibiting chitinase activity, and interfering with glycosphingolipid metabolism. Concurrently, it enhances plasma membrane permeability, induces pore formation, and suppresses ergosterol biosynthesis, collectively leading to cytoplasmic leakage. Secondly, thymol targets mitochondria, causing a decrease or hyperpolarization of the MMP and resulting in mitochondrial dysfunction. Furthermore, thymol triggers substantial intracellular accumulation of ROS and elevated Ca^2+^ levels, leading to oxidative stress and disrupting calcium homeostasis. These multifaceted damages ultimately converge to induce potassium ion efflux, further promote ROS accumulation, and trigger cytochrome C release, thereby activating the caspase cascade (e.g., Caspase-9 and its yeast homolog YCA1). This process is accompanied by the upregulation of key pro-apoptotic factors (such as YCA1, DNM1, NUC1, NDI1, and MMI1) and downregulation of anti-apoptotic regulators (such as FIS1 and CDC48), culminating in DNA fragmentation and systematically driving fungal cells toward programmed cell death.

Overall, the antifungal mechanism of thymol involves a multi-target synergistic cascade. It initiates by disrupting both the cell membrane (via ergosterol inhibition and pore formation) and cell wall (through interference with chitin and glycosphingolipid metabolism), compromising structural integrity. These primary damages trigger intracellular ion imbalance (e.g., K^+^ efflux and Ca^2+^ elevation) and oxidative stress (ROS production). Subsequently, mitochondrial dysfunction occurs, characterized by membrane potential collapse and cytochrome C release, which ultimately activates the caspase cascade and modulates apoptosis-related genes, leading to programmed cell death. A schematic diagram to illustrate the multi-target synergistic effects and cascade pathways of thymol’s antifungal action is shown in Figure 2.

### 3.5. Comparative Analysis of Antifungal Efficacy Between Thymol and Other Monoterpenoids

Besides thymol, several other natural monoterpenoids also exhibit antifungal activity, such as carvacrol, linalool, D-limonene, and eucalyptol. However, each has certain limitations compared to thymol. Carvacrol, an isomer of thymol, shows comparable antifungal activity but is more toxic [23]. Linalool and D-limonene are only effective against specific fungi and lack broad-spectrum activity [106,107]. In contrast, eucalyptol possesses relatively weak antifungal activity and is commonly used in synergistic antifungal applications [108]. The comparative antifungal activities of thymol and other monoterpenoids are systematically summarized in Table 3.

**Table 3:** Comparative analysis of antifungal efficacy between thymol and other monoterpenoids.

Compound	Chemical Class	Major Natural Sources	Core Mechanism of Action	Characteristics and Status	Reference
Thymol	Phenolic monoterpene	Thyme	Multi-target synergy: disrupts cell membrane and wall, inhibits ergosterol biosynthesis, induces mitochondrial damage and oxidative stress	Broad-spectrum, highly potent representative; most extensively studied	[38,85,93]
Carvacrol	Phenolic monoterpene	Thyme	Similar to thymol	Synergistic with thymol; higher toxicity compared to thymol	[23,109,110]
Linalool	Alcoholic monoterpene	Lavender	Disrupts cell membrane structure, inhibits hyphal growth	Particularly effective against specific molds (e.g., *Aspergillus flavus*)	[106]
D-Limonene	Hydrocarbon monoterpene	Citrus	Membrane interference, induces mitochondrial dysfunction	Shows significant activity against certain yeasts	[107]
Cineole (Eucalyptol)	Oxide monoterpene	Eucalyptus	Primarily membrane interference	Generally moderate to weak activity; often used in combination therapies	[108]

## 4. Treatment Strategies and Potential Clinical Significance

In contemporary medical applications, thymol has gained recognition as a versatile antifungal agent with broad utility. Studies on Thymus vulgaris essential oil and thymol have confirmed its inhibitory action on *Candida albicans* growth [41]. Wang et al. further demonstrated that thymol not only exhibits direct antifungal effects against *Aspergillus fumigatus* but also provides defense against corneal infections by inhibiting the LOX-1/IL-1β signaling cascade, which minimizes neutrophil and macrophage infiltration [40].

Additionally, integrating thymol into antifungal protocols demands careful consideration of its properties and potential synergies. Advanced delivery systems, such as starch Pickering emulsions for oral candidiasis, improve thymol’s bioavailability and activity against C. albicans when combined with enzymes like α-amylase, ensuring stability over weeks [111]. Chemical modifications, including sulfonamide group additions, have markedly increased thymol’s antimicrobial potency against pathogens like *Phytophthora capsici* and *Aspergillus flavus*, outperforming commercial fungicides such as azoxystrobin and carbendazim [112]. Synthesized derivatives, such as naphthoquinone/thymol hybrids via Williamson ether synthesis and copper-catalyzed reactions, bind to fungal ergosterol. This disrupts potassium permeability, causing ionic imbalance and cell death, with efficacy comparable to thiabendazole against *Fusarium solani* [113]. Formulations combining thymol with other agents, like fluconazole-loaded biopolymer films for resistant vaginal candidiasis, enhance drug distribution in tissues and boost anti-*Candida* effects, reducing fluconazole doses by 50% against resistant strains [114]. Combinations with caprylic acid disrupt cell membrane integrity and inhibit efflux pumps, leading to intracellular accumulation and mortality [115]. Additionally, thymol paired with Folicur^®^ effectively suppresses resistant *P. nodorum strains* [116], highlighting that integrated strategies can address resistant fungal infections effectively. Investigations into combination therapies reveal promising results; for instance, thymol synergizes with nystatin against Candida species in oral infections, reducing minimum inhibitory concentrations by 87.4% and achieving a fractional inhibitory concentration index of 0.25. This enhancement could lead to superior clinical outcomes, but comprehensive trials are essential to validate thymol’s standalone and combined efficacy in real-world scenarios [44]. This indicated thymol as a viable standalone or adjunct therapy for candidiasis in humans and animals.

## 5. Controversies and Challenges in Thymol’s Antifungal Use

### 5.1. Debates on the Efficacy of Thymol in Resistant Fungal Strains

The efficacy of thymol against resistant fungal strains is a topic of debate. While some studies have shown promising results, others suggest limitations. In the context of Candida species, which are known to develop resistance to conventional antifungal drugs, thymol has been tested against resistant strains. A study evaluated the activity of thymol in combination with fluconazole against clinical isolates of *Candida albicans*, *Candida glabrata*, and *Candida kruseii* [43]. Thymol, in combination with fluconazole, exhibited synergistic effects against all tested species, with FICI values ranging from 0.366 to 0.607 for *Candida albicans* strains, 0.367 to 0.482 for *Candida glabrata* strains, and 0.375 to 0.563 for *Candida kruseii* strains. This indicates that thymol can be effective against some resistant Candida strains when used in combination. Conte et al. demonstrated that a biopolymer film incorporating thymol combined with half the conventional dose of fluconazole exhibited potent fungicidal efficacy against drug-resistant Candida glabrata [114].

However, there is a lack of comprehensive studies on a wide range of resistant fungal strains. Different fungal species and strains may have varying susceptibilities to thymol, and the mechanisms of resistance may also play a role. For example, in bacteria, resistance mechanisms such as enzymatic inhibition, penicillin-binding protein modification, porin mutations, efflux pumps, and molecular modifications of antibiotic targets can affect the efficacy of antimicrobial agents [117]. Fungi may have similar or unique resistance mechanisms that could potentially reduce the effectiveness of thymol. More research is needed to fully understand the efficacy of thymol against resistant fungal strains and to develop strategies to overcome potential resistance issues.

### 5.2. Safety and Toxicity Concerns of Thymol in Clinical Use

Safety assessment of thymol reveals an application- and dose-dependent profile, with a generally favorable safety margin observed across multiple routes of exposure. When used as a poultry feed additive, thymol is classified by the U.S. Food and Drug Administration as Generally Recognized as Safe. Pharmacokinetic studies indicate that thymol is rapidly and completely absorbed in the gastrointestinal tract, exhibits a short half-life, and is primarily excreted via urine, thereby minimizing the risk of long-term accumulation [118]. Studies have confirmed its absence of neurotoxicity while demonstrating neuroprotective effects in cortical neurons [27,119,120]. Thymol induces apoptosis in A549 cancer cells while exhibiting no significant cytotoxicity toward normal peripheral blood mononuclear cells [121]. In broiler chickens, thymol administered at doses of up to 400 mg/kg demonstrated comparable protective efficacy to the synthetic antioxidant butylated hydroxytoluene, with no adverse effects reported [122]. When co-administered with carvacrol at doses of up to 200 mg/kg, thymol not only elicited no toxic effects but also enhanced antioxidant enzyme activities and improved immune responses, thereby promoting overall health [123]. However, thymol exhibits a narrow level of safety in anesthesia applications for zebrafish, i.e., low concentrations (50–75 mg/L) safely and effectively induce anesthesia with no mortality, and higher concentrations (≥100 mg/L) exhibit behavioral aversion and cardiorespiratory depression, with a 60% mortality rate observed at 200 mg/L, underscoring its significant toxic risk at elevated doses [124]. In chicken embryos, thymol demonstrated significantly lower toxicity compared to its isomer carvacrol, inducing only minor malformations at high doses without affecting embryonic growth [125]. Furthermore, thymol exhibits negligible estrogenic activity and mutagenic potential, with only inconsistent weak effects observed at very low concentrations that do not reach established safety risk thresholds [125]. A six-month chronic inhalation toxicity study further established that even prolonged exposure of mice to thymol at doses up to 858-fold the maximum human dose revealed no toxicological alterations in pulmonary and respiratory tissues [126]. More clinical studies are required to fully assess the safety and toxicity of thymol, especially in long-term use and in different patient populations.

### 5.3. Regulatory Challenges in the Approval of Thymol-Based Treatments

The approval of thymol-based treatments faces several regulatory challenges: (i) It lacks comprehensive data on its safety, efficacy, and long-term effects. Regulatory agencies typically require extensive pre-marketing approval data, including detailed toxicology studies, clinical trials, and information on the manufacturing process to ensure the quality, safety, and effectiveness of the treatment. For thymol-based treatments, there is a need for more well-designed clinical trials to demonstrate their efficacy in treating specific fungal infections. (ii) The standardization of thymol-based products is crucial. Natural products can vary in their composition depending on factors such as the source plant, extraction method, and formulation. This variability can make it difficult to establish consistent quality control and regulatory standards. For example, in the case of essential oils containing thymol, the concentration of thymol and other components can vary significantly, which may affect the product’s efficacy and safety. Regulatory agencies need to develop clear guidelines for the standardization of thymol-based treatments to ensure their reliable and reproducible performance. Moreover, the potential interactions of thymol with other medications also need to be investigated and regulated to avoid adverse drug–drug interactions in clinical use.

## 6. Future Directions and Innovations in Thymol’s Antifungal Research

### 6.1. Emerging Technologies in Thymol Delivery Systems

Emerging technologies in thymol delivery systems offer promising opportunities to enhance thymol’s antifungal efficacy. One such technology is the use of nano-encapsulation. For example, nanoemulsions can improve the solubility, stability, and bioavailability of thymol. A study on the nanoencapsulation of a formulation based on thymol, methyl cinnamate, and linalool (Ne-TML) showed that it had excellent antifungal and anti-aflatoxin B_1_ potential [127]. The Ne-TML was able to completely inhibit the growth and Aflatoxin B1 (AFB_1_) production of fungi at low concentrations. Nanoencapsulation can protect thymol from degradation, control its release, and improve its penetration into fungal cells, thereby enhancing its antifungal activity.

Another emerging technology is the development of responsive delivery systems. For instance, enzyme-responsive food packaging systems based on pectin-coated poly (lactic acid) nanofiber films have been fabricated to load and release thymol [128]. These films can respond to the presence of enzymes secreted by microorganisms from food contamination, triggering the release of thymol. This targeted release mechanism can improve the efficiency of thymol in inhibiting fungal growth in food products, reducing the need for high-dose or continuous application. Such technologies can be translated to clinical applications, where responsive delivery systems could potentially improve the treatment of fungal infections by delivering thymol precisely to the site of infection.

### 6.2. Prospective Studies on Thymol’s Long-Term Antifungal Effects

Prospective studies on thymol’s long-term antifungal effects are essential to fully understand its potential as a therapeutic agent. Long-term studies can help determine the durability of thymol’s antifungal activity, its impact on fungal resistance development, and its safety in extended use. For example, in the treatment of recurrent fungal infections, understanding how thymol performs over an extended period is crucial. A study on the long-term suppressive therapy of fungal infections in patients with artificial implants using fluconazole showed the importance of long-term data [129]. Similarly, for thymol, long-term studies could evaluate its effectiveness in preventing recurrence of fungal infections, especially in immunocompromised patients who are at a high risk of repeated infections.

In addition, studies have also investigated the impact of thymol on the host’s microbiota. Fungal infections often occur in the context of a disrupted microbiota, and long-term use of antifungal agents can further affect the balance of microbiota. Prospective studies could explore how thymol affects the composition and function of the host microbiota over time and whether it has any beneficial or adverse effects on the overall health of the host. This information is vital for developing sustainable and effective antifungal treatment strategies using thymol.

### 6.3. Potential for Thymol in Combination Therapies for Fungal Infections

Combination therapies using thymol have significant potential in the treatment of fungal infections. Thymol has been shown to exhibit synergistic effects with various antifungal agents. This synergistic effect can potentially reduce the required dosage of individual drugs, minimizing the risk of toxicity and resistance development. In addition, combination therapies can target multiple aspects of fungal pathogenesis. Fungi often have multiple virulence factors and resistance mechanisms, and combination therapies can simultaneously target different pathways. For instance, thymol combined with agents that target different stages of biofilm formation or fungal cell metabolism could be more effective in treating biofilm-associated fungal infections. Future research could focus on identifying the most effective combinations, understanding the mechanisms of synergy, and optimizing the dosing regimens to maximize the therapeutic benefits of thymol-based combination therapies for fungal infections.

## 7. Conclusions

Thymol has strong antifungal activity, and its underlying molecular mechanisms involve cell membrane rupture, interference with cell wall synthesis, disruption of mitochondrial function and energy metabolism, inhibition of biofilm, inhibition of virulence factor expression, inhibition of key enzymes, and induction of cell apoptosis. Additionally, it also exhibited good safety. In the future, more clinical research for testing the therapeutic effect of thymol against fungal infection in humans and animals is needed.

## Data Availability

Not applicable.

## Abbreviations

The following abbreviations are used in this manuscript:

AFB1	Aflatoxin B1
ATP	Adenosine Triphosphate
BHT	Butylated Hydroxytoluene
CAPA	COVID-19-associated Pulmonary Aspergillosis
CDC48	Cell Division Control protein-48
CNKI	China National Knowledge Infrastructure
DNA	Deoxyribonucleic Acid
DNM1	Dynamin-1
EC_50_	Half Maximal Effective Concentration
EST2	Expressed Sequence Tag-2
FDA	U.S. Food and Drug Administration
FICI	Fractional Inhibitory Concentration Index
FIS1	Fission-1
γ-CDTL	γ-Cyclodextrin Inclusion Complex of Thymol
HIV/AIDS	Human Immunodeficiency Virus/Acquired Immunodeficiency Syndrome
IL-1β	Interleukin-1 Beta
IPA	Invasive Pulmonary Aspergillosis
KCNAB	Potassium Channel Subunit Beta
LOX-1	Lectin-type Oxidized LDL Receptor 1
MAPK	Mitogen-Activated Protein Kinase
MIC	Half Maximal Effective Concentration
MMP	Mitochondrial Membrane Potential
MMI1	Mitochondrial Mediator of Inheritance 1
NDI1	NADH Dehydrogenase (Internal) 1
NUC1	Nuclease 1
ROS	Reactive Oxygen Species
YCA1	Yeast Caspase-1
